# Comparison of safety and effectiveness between 23-gauge and 25-gauge vitrectomy surgery in common vitreoretinal diseases

**DOI:** 10.1371/journal.pone.0248164

**Published:** 2021-03-05

**Authors:** Aleksandra Sedova, Irene Steiner, Rene Peter Matzenberger, Michael Georgopoulos, Christoph Scholda, Katharina Franziska Kriechbaum, Claudette Abela-Formanek, Georgios Mylonas, Stefan Sacu, Ursula Schmidt-Erfurth, Andreas Pollreisz

**Affiliations:** 1 Department of Ophthalmology and Optometry, Medical University Vienna, Vienna, Austria; 2 Center for Medical Statistics, Informatics and Intelligent Systems, Section for Medical Statistics, Medical University Vienna, Vienna, Austria; University of Toronto, CANADA

## Abstract

**Purpose:**

To assess and compare safety and effectiveness between 23-gauge and 25-gauge vitrectomy systems for the treatment of common vitreoretinal diseases in non-vitrectomized eyes.

**Methods:**

Retrospective evaluation of patients who underwent pars plana vitrectomy from April 2018 to December 2019 at the Department of Ophthalmology and Optometry at the Medical University of Vienna (MUV) for the following indications: macular epiretinal membrane, macular hole, macular lamellar hole, vitreous hemorrhage, vitreous opacities, vitreomacular traction syndrome and macular edema.

**Results:**

201 eyes of 195 patients that underwent 23-gauge (n = 105 eyes) or 25-gauge (n = 96 eyes) vitrectomy were included in this study. The mean best-corrected visual acuity (BCVA) improved at 1–3 months postoperatively and beyond 3 months in both gauge groups. Risk of any complication within 1 month postoperatively was lower in the 25-gauge group, but the difference was statistically not significant (HR [95% CI]: 0.95 [0.53; 1.70], p = 0.85). Intraocular pressure less than 5 mmHg was observed in 2 eyes (2%) in the 23-gauge group at the first postoperative day. Intraocular pressure elevation over 25 mmHg occurred in 5 eyes (2 eyes, 2%, in 23-gauge and 3 eyes, 3%, in 25-gauge group) at postoperative day 1, between 7 and 28 days in 5 cases (2 eyes, 2%, in 23-gauge and 3 eyes, 3%, in 25-gauge group), and in 2 eyes (2%) of the 23-gauge group at postoperative day 145 and 61, respectively. Retinal detachment occurred in 1 eye (1%) in the 23-gauge and in 3 eyes (3%) in the 25-gauge group. We did not observe any cases of endophthalmitis.

**Conclusion:**

Results in terms of safety, surgical success and visual outcomes for the treatment of common vitreoretinal surgery indications seem to be comparable between 23-gauge and 25-gauge vitrectomy systems, indicating that the two gauge systems can be used equally in the clinical routine.

## Introduction

Pars plana vitrectomy was developed by Robert Machemer in 1970 with the first vitrectomy devices consisting of a blunt hypodermic needle (17-gauge, 1.5mm) with a drill tip connected to a micromotor, which allowed aspiration, grasping and cutting of the vitreous [1–3]. In 1974, a new design for vitreous instruments was proposed by Conor O´Malley and Ralph Heintz resulting in 20-gauge (0.9mm) instruments with separate entries for vitreous cutter, infusion and illumination [4, 5]. This new approach required closure of sclerotomy sites with sutures after removal of the ports and proved to be successful [4, 5]. In 2002, Gildo Fujii et al. developed a 25-gauge (0.55mm) transconjunctival vitrectomy system [6]. A major disadvantage of the first 25-gauge instruments was the flexible instruments, which made surgery procedures more challenging. In 2005, Claus Eckardt introduced 23-gauge (0.72mm) sutureless vitrectomy instruments that, compared to the ones from Fujii et al., offer more stability due to their larger physical size [7]. The overall advantages of smaller vitrectomy systems are less risk for intraoperative and postoperative complications, increased postsurgical patient comfort and faster recovery times due to less traumatic intraocular access through smaller sclerotomies [6, 8].

Initially due to increased flexibility associated with smaller gauge instrumentation surgical indications were limited to cases not requiring extensive vitrectomy (e.g., peripheral retinal detachment) or complex epi- or subretinal membrane removal in the presence of proliferative vitreoretinopathy and included macular epiretinal membrane, macular hole, vitreomacular traction syndrome, or vitreous opacities [9–13].

In the last decades numerous technical improvements of 25-gauge instrumentation were implemented, which enhanced instrument rigidity, increased vitreous cutting rate and brightness of light sources [14, 15]. These modifications allowed safe application of 25-gauge instrumentation for more complex cases such as diabetic tractional retinal detachment, rhegmatogenous retinal detachment or dense vitreous hemorrhages [8, 15–19].

The aim of this retrospective study was to assess and compare the safety and effectiveness between 25-gauge and 23-gauge vitrectomy systems for the treatment of common vitreoretinal surgery indications except retinal detachment in nonvitrectomized eyes.

## Methods

### Subjects

After the approval of the Ethics Committee of the Medical University Vienna (MUV), a retrospective data analysis was performed on all patients who underwent vitrectomy from April 2018 to December 2019 at the Department of Ophthalmology and Optometry at MUV. All surgical reports and medical records of consecutive patients that underwent vitreoretinal surgery during this time period were reviewed by a vitreoretinal surgeon (A.P.). We employed strict inclusion criteria, which included primary vitrectomy with 23- or 25-gauge vitrectomy system for following indications: macular epiretinal membrane, macular hole, macular lamellar hole, vitreous hemorrhage, vitreous opacities, vitreomacular traction syndrome and macular edema. Exclusion criteria included history of any prior vitreoretinal surgery including retinal detachment surgery. Follow-up period was required for at least 4 weeks postoperatively. Data collected included age, sex, indication for surgery, surgical method, gauge-system, duration of surgery, type of anesthesia, lens status, refractive error, preoperative and postoperative Snellen best corrected visual acuity (BCVA), intraoperative and postoperative complications, induction of posterior vitreous detachment, number of sutures, intraocular pressure, number of follow-ups, single success surgery, second surgery. The surgery duration was defined as the period between insertion and removal of the lid speculum. Intraocular pressure was measured with Goldmann applanation tonometer. Ocular hypotony was defined as intraocular pressure of 5 mmHg or less. Ocular hypertension was defined as intraocular pressure of 25 mmHg or more.

For analysis of visual outcomes, we divided the patients into 2 treatment subgroups depending on whether an intravitreal gas tamponade was performed at the end of surgery.

Further, for analysis of surgery duration our patient cohort was subdivided into disease groups based on the need for epiretinal or inner limiting membrane (ILM) peeling, which typically extends surgery time and was performed in all eyes with macular epiretinal membrane, macular hole or macular lamellar hole but not in eyes with vitreous hemorrhage, vitreous opacities, vitreomacular traction syndrome or macular edema. The effect of cataract surgery on overall duration of surgery was adjusted in our statistical models.

### Surgical procedure

Patients received either general anesthesia (n = 148 eyes) or peribulbar block (n = 53 eyes). Eyelids and periocular skin were prepared with 5% polyvidone-iodine solution. Polyvidone-iodine drops were applied directly to the eye at the beginning and at the end of the surgery. Sclerotomies were performed at an oblique angle 3.5 mm to 4mm from the limbus in the inferotemporal, superotemporal, and superonasal quadrants. All patients were operated with the OS4 surgery system (Oertli Instruments, Berneck, Switzerland) applying the same settings (peristaltic pump) and either 23 or 25-gauge with a continuous flow cutter (double edged blade) and valved Oertli trocars. The choice of the vitrectomy system was up to the surgeon´s discretion. The surgical interventions and follow-up visits were performed by experienced vitreoretinal surgeons at the Department of Ophthalmology at MUV. Post-surgical treatment consisted of corticosteroid, non-steroidal inflammatory and antibiotic eye drops for 4 weeks.

### Statistical analysis

Quantitative variables are reported as mean ± standard deviation, if not stated otherwise. For qualitative variables absolute frequencies and percentages are reported.

The primary endpoint was the time from surgery until first complication within 28 days after surgery. Patients without complication were censored at the day of last observation or at day 28, respectively, if the observation period was equal or greater than 28 days. A mixed effects cox model (R-package coxme version 2.2.-16, R-function coxme) was calculated with patient as random factor. The interaction term with gauge system was also analyzed but removed from the model, since the p-value of the F-test was > 0.05 (results not shown). For group comparisons (23 gauge system, 25 gauge system) of surgery time (in minutes) and BCVA difference to baseline, mixed models were calculated (SAS Proc mixed) with patient as random factor. The degrees of freedom were calculated by the Kenward-Roger approximation. In the mixed model with surgery time as dependent variable, we adjusted for cataract surgery (no/ yes) and indication for vitrectomy. The interaction terms with gauge system were also analyzed but removed from the model, since the p-values of the F-tests were > 0.05 (results not shown). BCVA difference to baseline was analyzed for the follow-up category (28, 84] days and > 84 days and for patients with/ without gas tamponade separately. BCVA pre surgery and days from surgery until follow-up visit were the additional independent variables. For the analysis of BCVA, the entries counting fingers, hand motion and light perception have been set to 0.

Statistical analyses were conducted with R 3.6.2 and SAS 9.4. For all analyses, the significance level has been set to 0.05.

## Results

Two-hundred and one eyes of 195 patients undergoing vitrectomy were included in this retrospective study.

Out of the patients treated in one eye, 98 (47 female) were treated with 23 gauge and 91 (57 female) with 25 gauge instruments. Out of the 6 patients treated on both eyes, 2 patients were treated with 23 gauge on both eyes, 1 patient with 25 gauge on both eyes, 3 patients with 23 gauge on one eye and 25 gauge on the other eye. Time between surgeries of the two study eyes ranged between 39 days and 525 days. In the 23-gauge group, the mean age was 68.77 ± 9.52 years (age range: 37–90 years) and in the 25-gauge group 69.95 ± 9.56 years (age range: 28–87 years). The baseline characteristics are summarized in **Table 1**.

**Table 1:** Baseline characteristics of patients.

Characteristics	23-gauge (n = 105 eyes, 103 patients)	25-gauge (n = 96 eyes, 95 patients)
Age at time of surgery, years (mean ± SD)	68.77 ± 9.52	69.95 ± 9.56
Gender (male:female)	53:50	37:58
Eyes (right:left)	52:53	48:48
Preoperative BCVA (mean ± SD)	0.40 ± 0.26	0.40 ± 0.25
Lens, n (%)		
• phakic	57 (54%)	54 (56%)
• pseudophakic	48 (46%)	42 (44%)
Myopia, n (%)	54 (63%)	52 (59%)

This table shows the baseline characteristics of our study patients. As 3 patients were included with vitrectomy surgery performed on both eyes with different gauge systems the patients are listed in both columns.

The most common indication for vitrectomy in both groups was macular epiretinal membrane peeling (43% in 23-gauge and 64% in 25-gauge group) followed by macular hole (19% in 23-gauge and 15% in 25-gauge group). Other indications included macular lamellar hole, vitreous hemorrhage, vitreous opacities, vitreomacular traction syndrome and macular edema (**Table 2**).

**Table 2:** Indications for surgery.

Indication for vitrectomy	23-gauge (n = 105 eyes, 103 patients)	25-gauge (n = 96 eyes, 95 patients)
Epiretinal membrane, n (%)	45 (43%)	61 (64%)
Macular hole, n (%)	20 (19%)	14 (15%)
Vitreous hemorrhage, n (%)	17 (16%)	9 (9%)
Vitreomacular traction syndrome, n (%)	16 (15%)	1 (1%)
Macular lamellar hole, n (%)	5 (5%)	7 (7%)
Vitreous opacities, n (%)	1 (1%)	4 (4%)
Macular edema, n (%)	1 (1%)	0

This table shows the different retinal diseases included in our analysis subdivided by the gauge system used for surgery. The indicated percentages in each column refer to the respective gauge group.

Cataract surgery was combined with vitrectomy in 60 eyes (32 cases in 23-gauge and 28 cases in 25-gauge group).

All eyes with surgical indication of macular hole or macular lamellar hole were filled with 20% SF6 gas (n = 18 in 23-gauge and n = 16 in 25-gauge group). In addition, gas filling at the end of surgery was performed in eyes with a primary diagnosis of epiretinal membrane in 8 eyes of the 23-gauge group (7 eyes with 20% SF6 gas and 1 eye with air) and 10 eyes in the 25-gauge group (4 eyes with 20% SF6 gas and 6 eyes with air). In these eyes the presence of a macular hole was suspected (intraoperative OCT not available) based on surgical reports.

Silicon oil was used in 1 eye operated with the 23-gauge and in 1 eye with the 25-gauge system with surgical indication vitreous hemorrhage.

**Fig 1:**
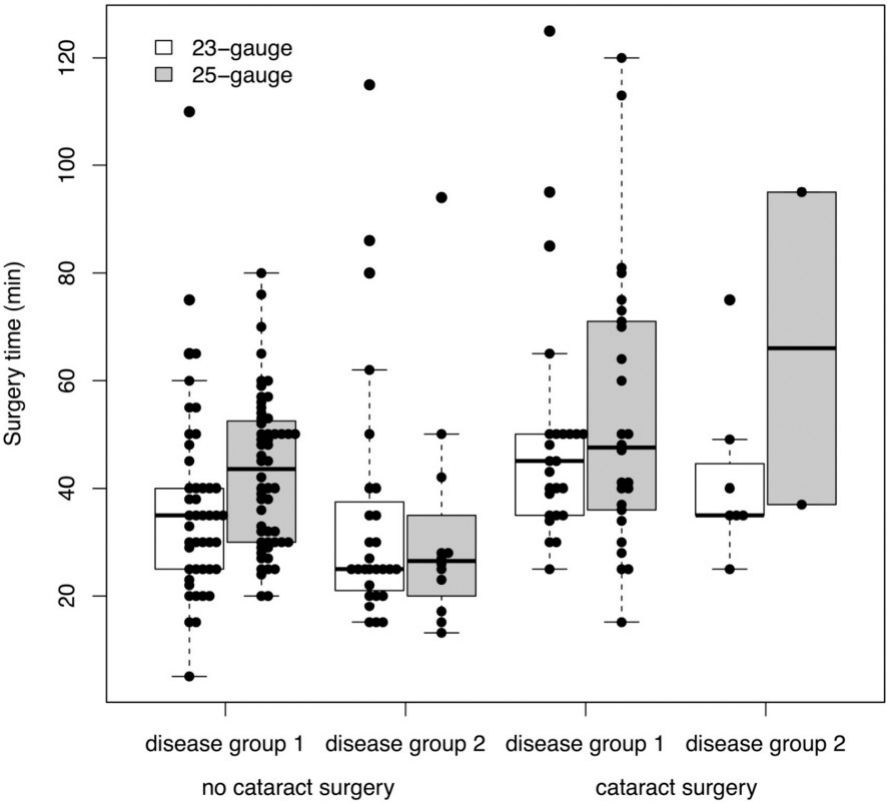
Surgery time. Boxplots and individual observations of surgery time in minutes. Disease group 1 = macular epiretinal membrane, macular hole, macular lamellar hole. Disease group 2 = vitreous hemorrhage, vitreous opacities, vitreomacular traction syndrome and macular edema. In the box plots, the inferior boundary of the box indicates the 25th percentile, a black line within the box marks the median, and the superior boundary of the box indicates the 75th percentile. Outliers are defined as values that are smaller/ greater than 1.5 times the interquartile range (IQR) from the box. Whiskers above and below the box indicate the minimum and the maximum, respectively, if no outliers are present. In case of outliers, the whiskers extend to the smallest/ largest value within the interval [25th percentile—1.5 IQR; 75thpercentile + 1.5 IQR].

The mean overall surgery duration was 39.66 ± 21.24 minutes in the 23-gauge group and 45.02 ± 20.53 minutes in the 25-gauge group. Mean surgery time did not differ significantly between the two gauge systems calculated with a mixed model with the mean group difference [95% CI] adjusted for cataract surgery and for the 2 disease groups (group 1: macular epiretinal membrane, macular hole or macular lamellar hole; group 2: vitreous hemorrhage, vitreous opacities, vitreomacular traction syndrome or macular edema): -2.96 [-8.79; 2.88], p = 0.32, see **Fig 1**.

No vitrectomy related intraoperative complications, such as lens touch, conversion to other gauge system or problems with surgical instrument functionality were observed in both gauge groups. The mixed effects Cox regression model revealed that the risk for a complication within the first month postoperatively was lower for the 25-gauge compared to the 23-gauge group, but the difference was not statistically significant (HR [95% CI] adjusted for indication: 0.94 [0.53; 1.70], p = 0.85).

In the 23-gauge group, 32 eyes (30%) needed suturing sclerotomy with 15 eyes (14%) requiring suture of one sclerotomy site, 4 eyes (4%) suture of two sites, and 13 eyes (12%) suture of all three sclerotomy sites. In the 25-gauge group, 30 eyes (31%) required suturing sclerotomy with 18 eyes (19%) requiring closure of one sclerotomy site, 5 eyes (5%) suture of two sclerotomy sites, and 7 eyes (7%) sutures of all three sclerotomies.

Two cases (2%) of hypotony were observed at the first postoperative day in the 23-gauge group, which resolved within 1 week without any intervention. Ocular hypertension was observed on the first postoperative day in a total of 5 cases (2 eyes, 2%, in 23-gauge and 3 eyes, 3%, in 25-gauge group) and between 7 and 28 days in 5 cases (2 eyes, 2%, in 23-gauge and 3 eyes, 3%, in 25-gauge group), which were due to cortisone response. After stopping local cortisone eye drops and application of pressure-lowering drops normotonia was achieved in all of these eyes within 1 to 2 weeks. In two eyes (2%) of the 23-gauge group ocular hypertension was first diagnosed at postoperative day 61 and 145, respectively and treated with local eye pressure lowering drops under which the pressure normalized.

Induction of posterior vitreous detachment was performed in 57 eyes (54%) in the 23-gauge and in 43 eyes (45%) in the 25-gauge group. In one eye (1%) in the 23-gauge group with already detached vitreous a retinal break was observed at day 4 postoperatively and treated successfully with a single session of laser coagulation.

The rate of retinal detachment in our study was 1% in the 23-gauge and 3% in the 25-gauge group (total of 4 out of 201 eyes; 1 eye in the 23-gauge group). These occurred within 28 days following surgery (4, 5, 6, 26 days, respectively). In all cases, no retinal breaks were observed during initial surgery upon routinely performed scleral indentation. All patients with retinal detachment underwent successful reattachment by a single surgery (cryocoagulation, gas filling).

Mild vitreous hemorrhage following surgery in cases with indication for surgery other than primary vitreous hemorrhage was observed in 10 eyes (7 eyes in 23-gauge and 3 eyes in 25-gauge group) within the first postoperative month. In all these cases intravitreal hemorrhages resolved spontaneously. See **Table 3** for the occurrence of postoperative complications.

**Table 3:** Postoperative complications.

Postoperative complication	23-gauge (n = 105 eyes, 103 patients)	25-gauge (n = 96 eyes, 95 patients)
Vitreous bleeding, n (%)	7 (7%)	3 (3%)
Retinal detachment, n (%)	1 (1%)	3 (3%)
Retinal tear, n (%)	1 (1%)	0 (0%)
Hypotony, n (%)	2 (2%)	0 (0%)
Ocular hypertension, n (%)	6 (6%)	6 (6%)
Endophthalmitis, n (%)	0 (0%)	0 (0%)
Subconjunctival bleeding, n (%)	11 (11%)	11 (11%)

This table shows the occurrence of postoperative complications subdivided by the gauge system used for surgery. The indicated percentages in each column refer to the respective gauge group.

No cases of endophthalmitis were observed among our patients in either group.

Single surgery anatomical success (SSAS) was achieved in 101 eyes (96%) in the 23-gauge and in 95 eyes (99%) in the 25-gauge group. All eyes without SSAS showed a persistent macular hole.

**Fig 2:**
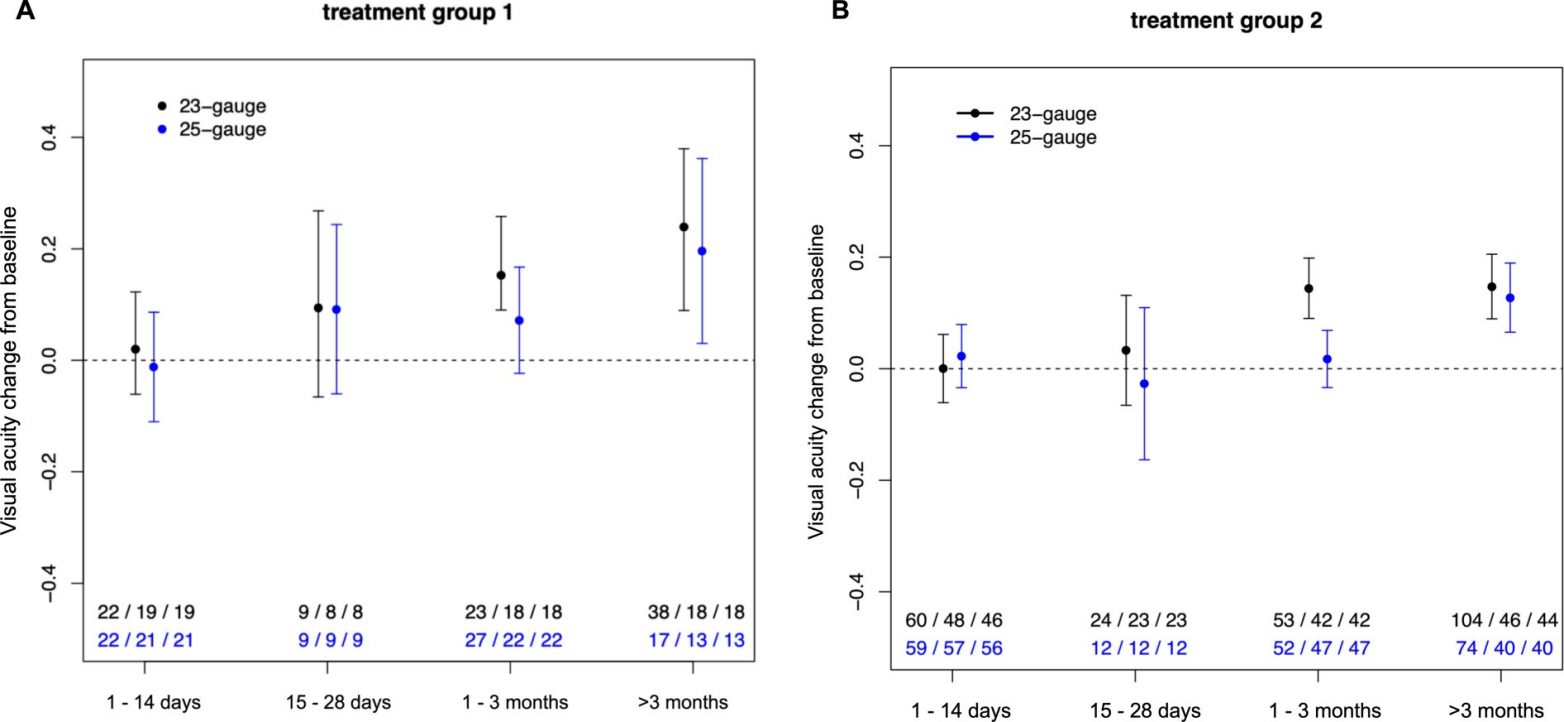
Visual acuity change from baseline. Mean visual acuity change from baseline subdivided by gauge system used for surgery (least squares means derived from the mixed models) with 95% confidence limits for patients of treatment group 1 (panel A: vitrectomy and gas tamponade) or group 2 (panel B: vitrectomy without gas tamponade). The numbers at the bottom of the figure show the number of observations / number of eyes / number of patients.

The preoperative BCVA (mean ± standard deviation) was 0.40 ± 0.26 in the 23-gauge and 0.40 ± 0.25 in the 25-gauge group. See **Fig 2A and 2B** for BCVAs changes.

In eyes treated with vitrectomy and gas tamponade overall BCVA at baseline was 0.30 ± 0.19 (23 eyes of 23 patients) in the 23-gauge and 0.30 ± 0.22 (25 eyes of 25 patients) in the 25-gauge group.

Within the subset of eyes that had a postoperative follow-up visit between 1 and 3 months, BCVA improved from 0.31 ± 0.20 at baseline to 0.43 ± 0.29 in the 23-gauge group (23 observations of 18 eyes / 18 patients) and from 0.28 ± 0.21 at baseline to 0.35 ± 0.23 (27 observations of 22 eyes / 22 patients) in the 25-gauge group. This difference of mean BCVA improvement between 23 and 25 gauge groups was statistically not significant (mean difference [95% CI]: -0.081 [-0.22; 0.054], p = 0.23).

Within the subset of eyes that had a follow-up visit beyond 3 months, BCVA markedly improved from 0.32 ± 0.20 at baseline to 0.47 ± 0.29 in the 23-gauge group (38 observations of 18 eyes / 18 patients) and from 0.28 ± 0.20 at baseline to 0.43 ± 0.33 in the 25 gauge-group (17 observations of 13 eyes / 13 patients). Again, this difference of mean BCVA compared to baseline between the two gauge systems was not statistically significant (estimate [95% CI]: -0.044 [-0.26; 0.17], p = 0.68).

In eyes treated without gas tamponade overall BCVA at baseline was 0.40 ± 0.29 (70 eyes of 68 patients) in the 23-gauge and 0.38 ± 0.24 (65 eyes of 64 patients) in the 25-gauge group. Within the subsets of eyes that had a follow-up visit between 1 and 3 months, BCVA improved from 0.39 ± 0.32 at baseline to 0.50 ± 0.28 in the 23-gauge group (53 observations of 42 eyes / 42 patients) and from 0.40 ± 0.24 at baseline to 0.43 ± 0.27 in the 25-gauge group (52 observations of 47 eyes / 47 patients). Within this timeframe BCVA increase between the 2 gauge systems was significantly lower for 25 compared to 23-gauge (mean difference [95% CI]: -0.13 [-0.20; -0.052], p = 0.0011). Within the subset of eyes that had at least one follow-up visit beyond 3 months, BCVA improved from 0.38 ± 0.26 at baseline to 0.54 ± 0.29 in the 23-gauge group (104 observations of 44 eyes / 44 patients) and from 0.33 ± 0.24 to 0.45 ± 0.26 in the 25-gauge group (74 observations of 40 eyes/ 40 patients). Again, no statistically significant differences were found when comparing the two gauge systems for the long-term follow-up visual acuity results (estimate [95% CI]: -0.020 [-0.10; 0.065], p = 0.64). In the postoperative time period 2 eyes in the 23-gauge and 5 eyes in the 25-gauge group underwent cataract surgery in the study eye.

## Discussion

In this retrospective study we evaluated safety and effectiveness of 25-gauge compared to 23-gauge vitrectomy systems in patients suffering from common vitreoretinal diseases requiring surgical repair for macular epiretinal membrane, macular hole, lamellar macular hole, vitreous hemorrhage, vitreous opacities, vitreomacular traction syndrome or macular edema. We demonstrated that rates of intra- or postoperative complications, single surgery success or long-term visual acuity outcomes between the two gauge groups were similar.

Sutureless vitrectomy is considered a safe and effective procedure, but may in some cases result in complications, such as vitreous hemorrhage, hypotony, retinal tear, retinal detachment or endophthalmitis [8, 15–19]. In our study, postoperative complication rates after vitrectomy were not significantly different between the 23 and 25-gauge groups. Among eyes without primary vitreous hemorrhage as indication for surgery, 7% of eyes in the 23-gauge and 3% of eyes in the 25-gauge group showed postoperative vitreous rebleeding, which resolved spontaneously. Kumar et. al reported 4% of secondary bleeding after 23-gauge vitrectomy, while Nagpal et. al reported 3% in the 23-gauge and 13% in the 25-gauge group [14, 15]. In our study, 1% in the 23-gauge and 3% in the 25-gauge group developed retinal detachment postoperatively and 1% in the 23-gauge group showed an iatrogenic retinal break on the 4^th^ postoperative day. Sandali et al. reported that 0.5% of eyes undergoing surgery for epiretinal membrane peeling developed rhegmatogenous retinal detachment after surgery in the 20-gauge group, but not in 23-gauge and 25-gauge groups [11]. Also they reported a 6% rate of retinal tears in the 23-gauge group compared to a 2.2% rate in the 25-gauge group. Byeon et al. followed eyes after 25-gauge vitrectomy and observed retinal detachments in 6% of cases [20]. A large retrospective chart review of 579 eyes that underwent vitrectomy for different indications showed a retinal detachment rate of 8.2% in the 23-gauge and 4.2% rate in the 25-gauge group [21]. In a study of 92 patients conducted by Gupta et. al a retinal tear in 1 eye (1.1%) after 23-gauge vitrectomy was reported [22].

Endophthalmitis is a rare but serious complication after vitrectomy. A meta-analysis performed by Oshima et al. with 77956 cases showed a rate of 0.08% after 23-or 25-gauge surgery [23]. A similar endophthalmitis rate of 0.085% was reported in a recently published large retrospective study by Weiss et al. including 18886 cases of 23-gauge, 25-gauge and 27- gauge pars plana vitrectomy [24]. In our study no cases of acute infectious postoperative endophthalmitis were observed. Similar to previous studies with 25-gauge vitrectomy systems [12, 20], we observed no difficulties with instrument functionalities and no conversion to another gauge system was necessary during the surgical procedures.

One of the advantages of transconjunctival sutureless vitrectomy systems is quicker patient rehabilitation due to lower levels of suture related discomfort, inflammation and postoperative astigmatism [25, 26]. However, in eyes with suspicious wound leakage sclerotomy sutures are necessary. In our study 30% of eyes in 23-gauge group and 31% of eyes in 25-gauge required suturing sclerotomy with 8–0 Vicryl sutures after removal of the trocars. Postoperative hypotony is associated with an increased risk of endophthalmitis due to the possibility of intraocular access for microorganisms and an increased possibility of choroidal detachment [12]. Gupta et al. reported a rate of 7% of eyes requiring sclerotomy sutures after 25-gauge vitrectomy [27]. In contrast to our results several studies reported a higher rate of suturing sclerotomies in the 23-gauge compared with the 25-gauge group, but similar rates of 1 day postoperative hypotony [8, 15, 26]. One possible explanation for the high suture rate in our patient cohort could be low surgeon’s tolerance if any wound leakage was observed, which may also explain the low rate of postoperative hypotony observed. Postoperative hypotony has previously been described as a common complication with an incidence ranging from 0.9% to 17% [11, 28–31]. Among our patients only in 2% of eyes hypotony (IOP = 4 mmHg) was observed 1 day after sutureless surgery performed with a 23-gauge vitrectomy system for epiretinal membrane peeling in one case and for vitreous hemorrhage in another. 2% of eyes in 23-gauge and 3% of eyes in 25-gauge group presented with IOP elevation over 25mmHg in the first postoperative day. However, one eye received SF6 gas intravitreally, one eye silicon oil and another intravitreal triamcinolone. Similar to the study conducted by Kim et. al, no significant differences in the occurrence of postoperative intraocular pressure elevation or hypotony were noted between 23-gauge and 25-gauge groups [26]. A previous study performed by Nam et al. showed no significant differences in visual recovery and postoperative inflammation between 25-gauge and 23-gauge vitrectomy groups among patients with various vitreoretinal diseases [8]. Surgical procedures were performed by experienced vitreoretinal surgeons at the Department of Ophthalmology of MUV with the same vitrectomy system (OS4; Oertli, Berneck, Switzerland) and the same settings using the peristaltic pump. In our study the mean surgery time did not differ significantly between the two gauge systems. Kumar et al. noticed among patients operated for diabetic macular traction retinal detachment that the mean surgical time in the 25-gauge group was significantly longer compared to the 23-gauge group [15]. Similar to our results, in a study conducted by Sandali et al. on patients treated for epiretinal membranes with different gauge systems the mean surgical time was not significantly different between 23-gauge and 25-gauge groups [11].

We showed a similar macular hole closure rate (96% in 23 gauge and 99% in 25-gauge group) after macular hole surgery compared to other studies with results ranging from 93 to 100% [32–35].

The mean BCVA was improving significantly less in the subgroup without gas infusion in the 25-gauge group compared to the 23-gauge group between month 1 and 3 postoperatively, for which we cannot provide any clinically plausible explanation. Importantly, after month 3 this difference was not statistically significant anymore. Sandali et al. showed BCVA improvement 6 weeks postoperatively after epiretinal membrane surgery, which was not significantly different between 25 and 23-gauge groups. However, BCVA improvement was higher in the 25-gauge group on the 8^th^ postoperative day, compared to 23-gauge group, as well as when compared to the 23-gauge group with and without sutures [11]. Gupta et al. demonstrated an overall BCVA improvement after 23-gauge vitrectomy as well as when divided by surgical indication (retinal detachment, vitreous hemorrhage, epiretinal membrane and macular hole) [22].

Limitations to our study are characteristic of a retrospective study design with different follow-up duration and loss of follow-up. The limited number of patients for the time period indicated is due to the strict inclusion criteria with a follow-up period of at least 4 weeks. As the Department of Ophthalmology at MUV is a tertiary referral center for complex retinal surgical cases the number of routine vitreoretinal surgery cases, which met inclusion criteria for this study, is limited. Further, a number of patients referred from other hospitals typically have their follow-up visits after the 1-week postoperative check-up at their referring eye center for travel distance reasons excluding them for inclusion in this study. Due to the retrospective study character the use of different gauge systems was not equally balanced between the vitreoretinal surgeons with four out of seven having performed more 25-gauge and the remaining three mainly 23-gauge cases. Further, retinal detachment cases were not included as the majority of these surgeries were performed with the 23-gauge system causing a major imbalance between the gauge systems in the analysis.

Strengths of this retrospective study include the selection of the study population by a vitreoretinal specialist through detailed review of medical charts and well-balanced patient groups with subgroup analyses for visual acuity outcome and surgery duration. Further, use of the same surgical platform with similar settings by experienced vitreoretinal surgeons benefits the comparability of data between the gauge systems.

In conclusion our retrospective analyses of vitreoretinal surgical cases at the Department of Ophthalmology at MUV revealed that 23-gauge and 25-gauge vitrectomy systems seem to be comparable in terms of intra- and postoperative safety, surgery time and long-term visual acuity outcomes. These results suggest that 25-gauge vitrectomy systems in the clinical routine can be used equally to 23-gauge systems in common indications for vitreoretinal surgery.
